# Correction: The UVSSA complex alleviates MYC-driven transcription stress

**DOI:** 10.1083/jcb.20180716302092023c

**Published:** 2023-02-23

**Authors:** Mai Sato, Rowyn C. Liebau, Zhaoqi Liu, Lizhi Liu, Raul Rabadan, Jean Gautier

Vol. 220, No. 2 | 10.1083/jcb.201807163 | January 6, 2021

The author line in this article has been updated to reflect an author name change. Andrew W. Liebau has been changed to Rowyn C. Liebau.

